# The Perspective of a Breast Cancer Patient: A Survey Study Assessing Needs and Expectations

**DOI:** 10.7759/cureus.9171

**Published:** 2020-07-13

**Authors:** Amulya Prakash, Muhammad Sardar, Nasreen Shaikh, Sindhura Inkollu, Mary Danish, David J Sharon, Shira Goldberg

**Affiliations:** 1 Internal Medicine, Monmouth Medical Center, Long Branch, USA; 2 Oncology, Monmouth Medical Center, Long Branch, USA; 3 Oncology, Monmouth Medical Center, Leon Hess Cancer Center, Long Branch, USA; 4 Hospice and Palliative Care, Monmouth Medical Center, Long Branch, USA

**Keywords:** breast cancer research, breast cancer outcomes, health communication, psycho-oncology, integration of palliative care service

## Abstract

Background

Patient satisfaction is one of the key indicators of health care quality. We aim to identify patient's needs and expectations in a breast cancer clinic to provide patient-centered care and better overall satisfaction.

Methods

A 17-item survey was administered to 110 patients at a breast cancer clinic. The survey was designed after a thorough literature review and approved by an oncologist and a palliative care physician.

Results

Self-reported knowledge about the disease was reported adequate by 90.9% of our patients yet only 55.45% of our patients could identify the stage of their cancer. More education was desired by 32.7% of patients including various treatment options (29%), common complications (24.5%), prognosis (26.3%) and risk factors (11.8%). The majority of our patients were having some form of cancer-related emotional stress and physical symptoms. The majority of our patients (57.27%) wanted their oncologist to address social/emotional issues and 25.45% felt the need for more focus on physical symptoms in their subsequent visits. End-of-life (EoL) care discussions were considered an integral component of overall care by 29% of our patients. Components of EoL care discussions that patients stated they could benefit from included prognosis (27.27%), life expectancy (29%), the treatment effect on the quality of life (22.7%), palliative care (9%), hospice (10.9%), advance directives (11.8%), and family involvement in medical decision-making (13.6%). There was a difference noted regarding their EoL care discussion based on the stage of cancer. Patients with early-stage disease wanted their oncologists to decide on the frequency of this discussion (72.7%). Patients with advanced disease wanted EoL care discussion to be done more frequently as initiated by them or their oncologist or if there’s a change in the treatment plan.

Conclusions

A discrepancy between self-reported and actual knowledge in breast cancer patients emphasizes the need for patient education. Most patients rely on their oncologists for their diagnosis-related emotional and social issues. Surprisingly, more than a quarter of our patients consider EoL care discussions important even though the majority of our patients were healthy and having stage I and II disease.

## Introduction

Breast cancer is the most common cancer among women in the United States and worldwide, excluding non-melanoma skin cancers. As per the National Cancer Institute, estimated new cases of female breast cancer in 2019 were 268, 600 which is 15.2% of all new cancer cases in the United States. The lifetime risk of developing this cancer in a woman is approximately 12.8% at some point during their lifetime, based on the data from 2014-2016 [1]. However, with recent advancements in screening methods, diagnostic modalities, and treatment options, relative survival has improved from 74.6% in 1975-1979 to 90.6% in 2006 [2].

Diagnosis and treatment of breast cancer patients are complex with common principles modified by patient's unique issues. Breast cancer patients may be broadly divided into three groups: a) those with early-to-advanced-stage disease, i.e., those for whom adjuvant therapies may be offered, b) those with metastatic disease and c) those who are breast cancer survivors (BCS). 

Patient's experience of breast cancer, throughout the continuum of their disease, will influence their "quality of life" [3]. This will be strongly influenced by their underlying understanding of their diagnosis, treatment, and prognosis. It is to that end that we devised the questionnaire utilized in this study. We attempted to better elucidate patient's knowledge, understanding and insight of their disease, and its treatment, and the impact it has had on their physical and psychological health. Our goal as health care providers (HCP) is to enhance the quality of our patient's medical as well as psychological outcomes. To that end, an enhanced understanding of our patients' underlying insights into their disease will guide us to their improved psychological well-being. At the same time, a well-informed patient would be able in a better way to voice their concern and guide their provider in their care. The first step, therefore, in this process, is to get a "snapshot" of the degree of their understanding. 

## Materials and methods

**Figure 1:**
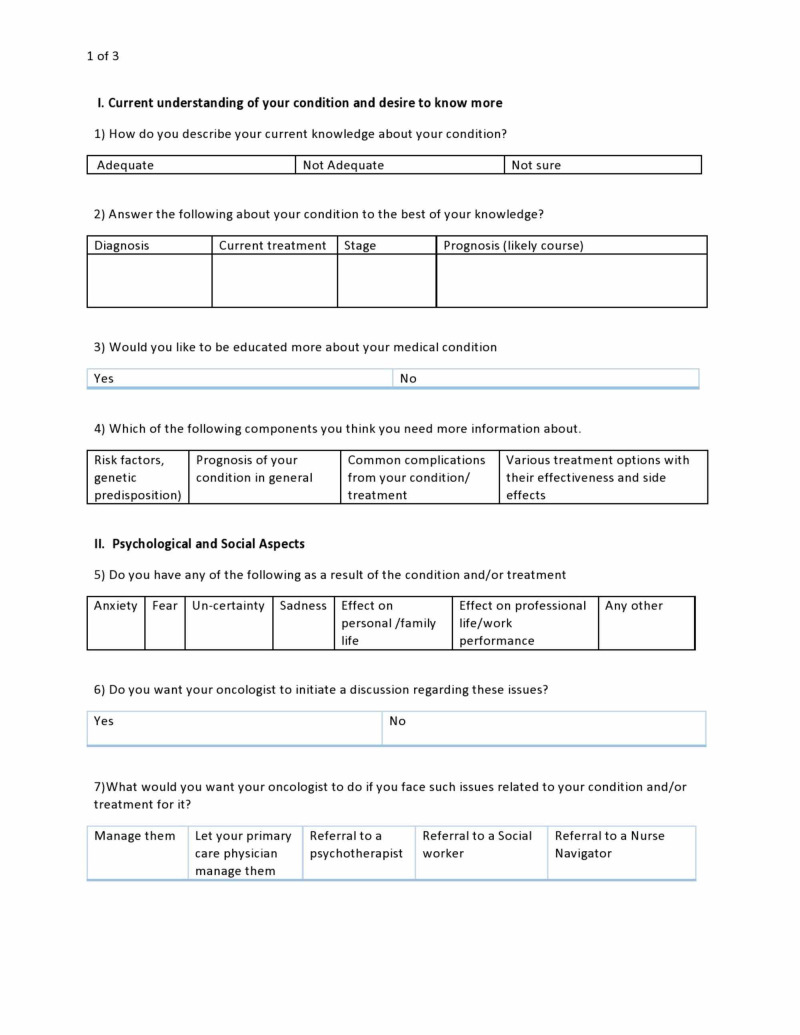
Sample survey questionnaire page 1

**Figure 2:**
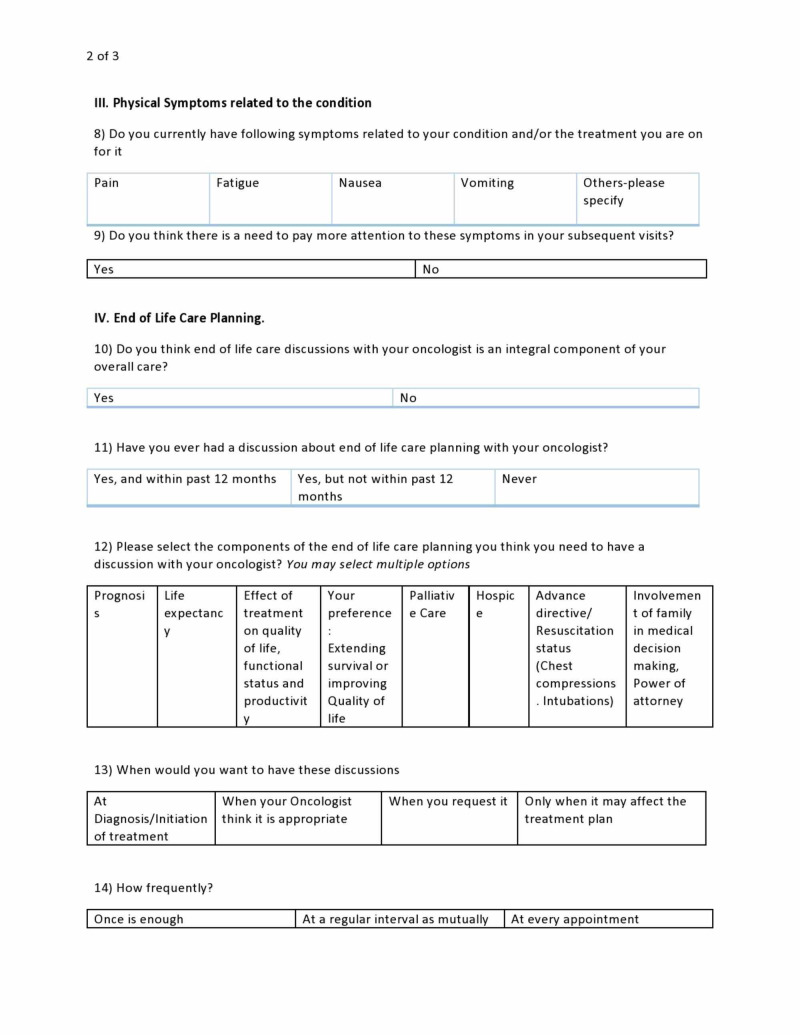
Sample survey questionnaire page 2

**Figure 3:**
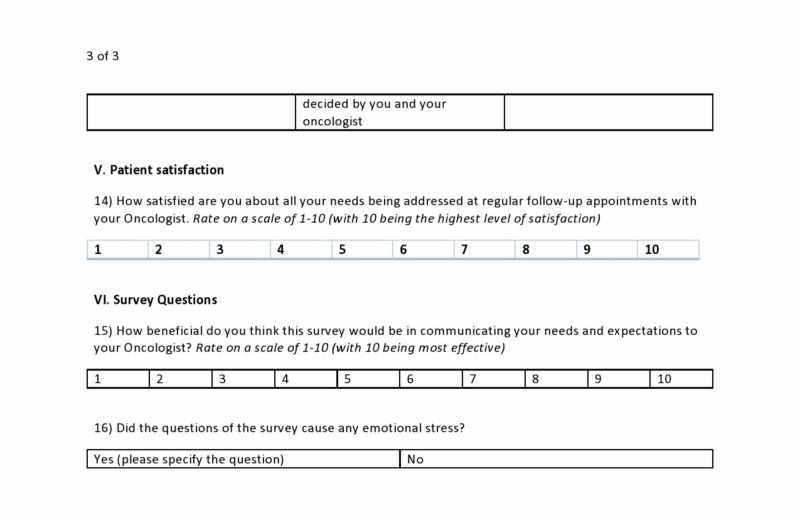
Sample survey questionnaire page 3

Patients were identified who were enrolled in our breast cancer clinic for treatment or follow-up. A 17-item survey was administered to 110 patients using a convenience sampling method over 12 months. The survey was designed after an extensive literature review with the help of an oncologist and a palliative care specialist and waived by Monmouth Medical Center Institutional Review Board (IRB). The questionnaire was designed to assess the patient's knowledge, the impact of diagnosis, and treatment on physical and psychological health, end of life (EoL) care planning (Figures 1-3).

Most of our patients were insured. The majority of them were English speakers in addition to other languages. Patients were approached by medical residents and research coordinator to fill out the survey. Nurse navigators were present to facilitate the process. Surveys were read out loud for patients with vision problems. Patients were also allowed to take the survey home and bring it back during the next visit.

## Results

For a detailed overview of the patient’s preference, see Table 1. Of all the included patients, 13.63% were stage 1, 13.63% were stage 2, 12.7% were stage 3, 16.36% were stage 4, 14.5% were BCS. 29.18% of total patients could not specify their disease stage. Patients who were referred for pre-cancerous lesions were excluded.

**Table 1:** Detailed result of the survey Please note all responses may not add up to exactly 100% because many patients chose not to answer or chose multiple answers in certain questions.

Serial No.	Items	Result^1^
I	Patient Characteristic	
Ia	Stage 1	13.63%
Ib	Stage 2	13.63%
Ic	Stage 3	12.7%
Id	Stage 4	16.36%
Ie	Breast cancer survivor	14.5%
If	Disease stage unspecified	29.18%
II	Current understanding of your condition	
1	How do you describe your current knowledge about your condition?	
1a	Adequate	90.9%
1b	Not adequate	6.4%
1c	Not sure	2.7%
2	Answer question related to your condition.	
2a	Able to record diagnosis	61.8%
2b	Current treatment	85%
2c	Stage	55.45%
2d	Aware of prognosis	
2d1.	Good	30.9%
2d2.	Bad	8.19%
2d3.	Not aware	59%
3	Would you like to be more educated?	
3a	Yes	32.7%
3b	No	64.5%
4	Which of the following components you think need more education about?	
4a	Risk factors and genetics	11.8%
4b	Prognosis	26.3%
4c	Complication	24.5%
4d	Treatment options and side- effects	29%
III	Psychological and social aspects	
5	Do you have any of the following as result of the condition and/ or treatment?	
5a	Anxiety	44.5%
5b	Fear	27.3%
5c	Uncertainty	27.3%
5d	Sadness	21.8%
5e	Effect on personal / family life	30.9%
5f	Effect on work performance	23.6%
5g	Other issues	1.8%
6	Do you want your oncologist to initiate discussions regarding above issues?	
6a	Yes	22.73%
6b	No	77.27%
7	What would you want your oncologist to do if you face such issues?	
7a	Manage them	57%
7b	Let your PCP manage	10%
7c	Referral to psychotherapist	9.01%
7d	Referral to social worker	11.8%
7e	Referral to nurse navigator	13.6%
IV	Physical symptoms related to your condition	
8	Do you currently have following symptoms related to your condition or treatment?	
8a	Pain	25.45%
8b	Fatigue	41.8%
8c	Nausea	18.18%
8d	Vomiting	4.54%
8e	Other	3.63%
9	Do you think there is a need to pay more attention to these symptoms in subsequent visits?	
9a	Yes	25.45%
9b	No	67.27%
V	End of life care planning	
10	Do you think end of life care discussions with your oncologist is an integral component of your overall care?	
10a	Response from patients in adjuvant treatment group (early to advanced stage disease)	
10ai	Yes	34.09%
10aii	No	65.91%
10b	Response from patient with metastatic disease	
10bi	Yes	44.5%
10bii	No	55.5%
10c	Response from breast cancer survivors	
10ci	Yes	43.75%
10cii	No	56.25%
11	Have you ever had a discussion about end of life care planning with your oncologist?	
11a	Yes, and within 12 months	4.5%
11b	Yes, but not within 12 months	10.9%
11c	Never	89%
12	Please select components (multiple) of the end of life care planning you think need to have discussion with your oncologist?	
12a	Prognosis	27.27%
12b	Life expectancy	26.36%
12c	Effect of treatment on quality of life	22.7%
12d	Your preference on extending survival vs quality of life	22.7%
12e	Palliative care	9%
12f	Hospice	10.9%
12g	Advance directive	11.8%
12h	Involvement of family in decision making	13.6%
13	When would you want to have these discussions?	
13a	Response from patients in adjuvant treatment group (early to advanced stage disease)	
13ai	At diagnosis/ initiation of treatment	6.8%
13aii	When oncologist think it’s appropriate	72.7%
13aiii	When you request it	15.9%
13aiv	When it may affect treatment plan	11.3%
13b	Response from patients with metastatic disease	
13bi	At diagnosis/ initiation of treatment	16.6%
13bii	When oncologist think it’s appropriate	44.44%
13biii	When you request it	44.44%
13biv	When it may affect treatment plan	11.11%
13c	Response from breast cancer survivor	
13ci	At diagnosis/ initiation of treatment	12.5%
13cii	When oncologist think it’s appropriate	50%
13ciii	When you request it	12.5%
13civ	When it may affect treatment plan	25%
14	How frequently?	
14a	Once	40%
14b	At regular interval mutually decided by patient and oncologist	32.7%
14c	Every appointment	5.4%

1. Self-reported knowledge by a patient

About 90.9% of our patients mentioned that they have adequate knowledge about their condition, 2.7% reported that they are not sure about their current understanding of the disease while 6.4% felt that their knowledge about their disease is not adequate. In actuality, only 55.45% could identify the stage of their cancer, and out of them, only 16.3% could provide further tumor characteristics. Interestingly, 85% of patients were aware of their treatment plans. 32.7% of our patients desired more education and information about their condition. 11.8% wanted to know more about risk factors, 26.3% about prognosis, 24.5% about complications, and 29% about various available treatment options.

2. Psychological and social aspects

Overall, 44.5% of patients suffered from anxiety, 27.3% were fearful about recurrence, and 27.3% had concerns related to uncertainty about their future; 23.6% of patients mentioned that their work is being affected because of their physical condition; 77.27% of our patients did not want their oncologist to initiate a discussion about these psychosocial issues. However, 57% of them expected their oncologist to manage those symptoms if already diagnosed and 10% of them wanted their primary care physician (PCP) to manage those symptoms.

3. Physical symptoms

Cancer-related fatigue was reported by 41.8% of patients, pain by 25.45%. Other symptoms that were commonly reported were nausea, joint pain, peripheral neuropathy, mouth sores, etc. For our cohort, 67.27% of patients mentioned that they did not feel the necessity for these symptoms to be discussed in subsequent visits, apparently, they were adequately managed.

4. EoL care planning

There were 34.09% of patients in the early stage of cancer, 44.5% in advanced stages of breast cancer, and 43.75% of BCS mentioned that they had EoL discussion with their oncologist. For those who had EoL discussion with their oncologist, they consider prognosis (27.27%), life expectancy (26.36%), the effect of treatment on quality of life, and functional status (22.7%) as the major topics to be focused on.

We noticed a difference between different patient groups regarding their preference for EoL care discussion. Among patient subgroups with early-to-advanced-stage disease on adjuvant treatment, 72.7% wanted this discussion at regular intervals decided by their oncologist, 6.8% wanted it at fixed intervals, 15.9% would prefer discussion when they desired and 11.3% would want it when there is a change in the treatment plan. In the BCS cohort, the majority of them (56%) also wanted to leave this discussion to their oncologist’s discretion. 

Patients with metastatic disease (18 patients) in response to EoL care discussion questions responded in the following fashion. 16.66% desire discussion at regular intervals, 44.44% desire discussion at oncologist discretion, 44.44% desire discussion when they want, 11.11% desire discussion at the time of change of therapy. As some patients responded to multiple choices, the percentage did not add up to 100%.

We asked our patients to rate the overall care they are receiving at our clinic on a scale of 1-10, while 1 being the worst and 10 being the best experience. We achieved an aggregate score of 9.22.

To adjudge the appropriateness of our survey, we asked our patients for a response as to whether they felt this survey might help improve their care and communication between their oncologists and them. We received an aggregate rating of 7.93 on the same scale of 1-10.

We also wanted to know if the survey or any of its components caused them any distress. 84.5% of patients were unbothered while 9% of them were distressed because of some survey questions, mostly related to EoL questions. 6.5% of total patients did not respond to this question.

## Discussion

In addition to the actual diagnosis of breast cancer, patient's knowledge of the specifics of their breast cancer such as stage, prognosis, therapeutic options can be critical to the quality of their journey through their disease. We encountered a disparity between actual knowledge and perceived knowledge in our patients. Patient's understanding of their condition modified goals of care and also defined their expectations from their HCP. With a surge in survivorship with the advancement in diagnosis and treatment of cancer, it is imperative that HCP understand the needs and perspectives of their patients of that they solicit the participation of their patients as well. There may be unidentifiable barriers that could limit their ability to relay their concerns. We designed a survey-based study to capture the concerns of these patients, their unmet needs, identify gaps in care, and improve overall patient satisfaction and outcomes. We also tried to analyze the problem from both the patient and physician perspectives.

As our study revealed, patients self-reported knowledge was high at 90.9% but their actual understanding of their disease, staging, and prognosis appeared less and therefore, contradictory. Our findings are consistent with prior studies [4]. Low educational state, low health literacy, ethnicity, the language barrier could all contribute but is beyond the scope of our research. Improving patient education may lead to better-informed decisions and better adherence to treatment plans. Studies have been previously done to evaluate if increased information access reduced distress in cancer patients [5]. As per Vogel et al. (2008), patients having breast cancer who were given information had a decreased level of anxiety and feeling of insecurity [6]. 80% of our cohort requested more information with the majority of them having concerns related to prognosis, future complications, and treatment options. Exploring further patient's concerns and providing supportive care could be attempted through various methods including internet-based education, telephone counseling, involving breast health specialists, and navigator in care [7,8].

We reached an intriguing conclusion about the role of oncologists in the psychosocial well-being of breast cancer patients. 77.27% of all our patients preferred their oncologist not to initiate discussion on psychosocial issues, however, 55.27% of the 77.27% would want their oncologists to address those issues if they already have been diagnosed with any psychosocial issues. What does that mean? Cancer patients may have multiple psychological issues that can go undetected or be attributed to adjustment, grief, or stress. Some patients who have lower cancer-related health education also tend to minimize their symptoms which can have quite a serious impact including depression, suicidal tendencies, refusal of treatment in advanced cases, etc. [8,9]. Patients might not request the additional provision of information when they have knowledge gaps. During the clinical encounter, clinical practitioners should assess the individual educational needs and tailor the education accordingly. Health care providers should continue assessing the informational needs of a patient during different stages of the treatment, realizing at the beginning patients’ and families can be overwhelmed by the information provided. This can lead to misunderstanding the information and new questions may emerge along the treatment process.

As there is generally a strong relationship between oncologists and patients, patients often presume their oncologist would address any symptoms related to the cancer diagnosis. Oncologists, on the other hand, may feel poorly equipped to handle general health issues or not related to cancer and may expect the participation of the PCP for those issues. It's quite possible that patients may confuse the role of their physicians. Our study emphasizes a need for better coordination of care among physicians. Lack of clear guidelines to the patient of which physician is responsible for which symptoms may compromise comprehensive care [10]. Practical tools such as the Hospital and Anxiety Depression Scale (HADS) and the Brief Symptom Inventory (BSI) have also been shown to be helpful to monitor psychological states across the disease trajectory [11]. However, this needs better coordination of care between HCPs. Some studies have shown that there is a disconnect between patient and physician, among physicians (PCP, oncologist, other subspecialties) as well which may lead to gaps in care [11-14].

Another spectrum of patient education is the EoL care discussion. Patients who are not well aware of their tumor staging and prognosis may fail to understand the importance of EoL care discussion and palliative care which may complicate care moving forward. As per one study, physicians tend to withhold information if a patient has a poor prognosis concerned that their patient may lose hope in treatment [15,16]. Patients who have a relatively lower level of understanding of their disease process may think in a similar fashion. Oncologists play a critical role in caring for a patient with cancer through treatment and in some cases to the EoL [16,17]. In our study, we found that only 44.5% of our patients with advanced disease wanted to have EoL discussion while 55.5% deferred it. Lack of proper cancer knowledge may lead to a lower desire for disease information due to a higher preference for a passive decision-making approach, being less autonomous in regards to decisions of medical care. EoL care discussion may provide a more realistic insight into their condition, especially in advanced cancer patients. It should include discussion of comprehension of prognosis, life expectancy, treatment options, side effects of treatment, quality of life. As per Saraiya et al. (2008), improvements in patient decision making and clinical practice can reduce the burden of symptoms for patients if clinicians gain a better understanding of patient's expectations respecting the longer-term consequences of diagnosis and treatment [18]. Early integration of palliative care in cancer improves symptom control, end-of-life care, health-related communication, and continuity of care [19].

Breast cancer patients often experience complications related to the disease itself, chemotherapy, and surgery. Cancer-related fatigue is very common and affects work and daily performance [20,21]. Patients on aromatase inhibitor frequently experience pain, musculoskeletal symptoms among other side effects [22]. Chronic arm lymphedema after breast surgery impairs physical performance and studies have shown that early mobilization and rehabilitation have improved outcomes [23]. It is imperative that the patient's physical symptoms be addressed early and adequately as physical disability often leads to depression [24].

Our study has certain limitations and strengths. It’s a single-center study with a relatively small study population. Our patients come from various ethnicities and backgrounds. However, we did not analyze our data in a way to accommodate cultural and ethnic variation in regards to their perspective towards disease and treatment. We propose a future multicenter study with a larger patient population with higher variation to validate our study. There can be a difference in patient perspective based on cancer type and prognosis which needs to be studied further. The final aim is to create a better method for the care of cancer patients tailored to their individual needs and eliminating gaps in care.

## Conclusions

Our study attempts to measure the level of awareness related to breast cancer diagnosis and its complications among our patients and highlights its impact on their overall care. Management of breast cancer is not limited to therapeutic measures only but also includes psycho-social wellness and EoL care discussion, particularly for patients with metastatic disease. The psychosocial aspect of management remains poorly understood in clinical practice and relatively neglected and that the cause of this is multifactorial. Lack of clear guidelines and disconnect between patients and physician and among physicians themselves contribute to the gap in care. Patient education will lead to an improvement in communication between patient and physician and eventually improve outcomes. Through our research, we also tried to understand patient perspective in their care. The bigger question that remains unanswered is how to increase patient’s involvement in their care at every step. We need better ways to improve the education, communication, and decision-making capacity of our patients. With the improvement in breast cancer survivorship, medical care of such patients is an emerging area for quality improvement.
